# TPPP3 promote epithelial-mesenchymal transition via Snail1 in glioblastoma

**DOI:** 10.1038/s41598-023-45233-w

**Published:** 2023-10-20

**Authors:** Xu Xu, Yunan Hou, Niya Long, Lishi Jiang, Zhangwei Yan, Yuan Xu, Ying Lv, Xin Xiang, Hua Yang, Jian Liu, Xiaolan Qi, Liangzhao Chu

**Affiliations:** 1 Department of Neurosurgery, The Affiliated Hospital of Guizhou Medical University, Guiyang, Guizhou China; 2 Guizhou Provincial People’s Hospital, Guiyang, Guizhou China; 3 Key Laboratory of Endemic and Ethnic Diseases, Ministry of Education & Key Laboratory of Medical Molecular Biology of Guizhou Province, Guizhou Medical University, Guiyang, Guizhou China

**Keywords:** Cancer, Cancer genetics

## Abstract

Tubulin polymerization promoting protein 3 (TPPP3), a member of the tubulin polymerization family, participates in cell progressions in several human cancers, its biological function and the underlying mechanisms in glioblastoma multiforme (GBM) remain unclear. Here, we investigated the role and application value of TPPP3 in gliomas and found that the expression of TPPP3 in glioma was higher than that in normal brain tissue (NBT), and increased with the grade of glioma. Up-regulation of TPPP3 expression in glioblastoma cells confer stronger ability of migration, invasion, proliferation and lower apoptosis in vitro. Inhibition of TPPP3 expression in GBM could reduce the migration, invasion, proliferation and induce the apoptosis of glioblastoma cells. TPPP3 affected the process of EMT by regulating the expression of Snail 1 protein. In clinical data analysis, we found a positive correlation between TPPP3 and Snail1 protein expression levels in glioblastomas. Low TPPP3 expression leads to better survival expectations in glioblastomas patients. The content of this study paves the way for further in-depth exploration of the role of TPPP3 in glioblastoma in the future, and provides new treatment and research directions.

## Introduction

Human glioma (Glioma) is a primary central nervous system malignant tumor that originates from neuroepithelial ectoderm in histological classification^1^. It ranks first in the global incidence and is also the most difficult intracranial tumor to overcome^2^. The abnormal proliferation ability, anti-apoptotic characteristics, and aggressive growth characteristics of glioma cells are difficult to cure glioma. Even during high-dose chemotherapy and radiotherapy, tumor cells can continue to infiltrate the surrounding brain tissue^3,4^. The mechanism of invasion of glioma cells is still unclear. High grade gliomas, especially glioblastoma multiforme (GBM), are common and highly malignant intracranial primary tumors in adults. Previous studies have shown that glioma cells infiltrate peripheral along the basement membrane of blood vessels or diffusely along channels between extracellular matrix, neurons and astrocytes^5^. Therefore, it is of great importance to study the biological characteristics and pathological mechanism of GBM and find new mechanisms and new perspectives to fight the growth and metastasis of GBM for the treatment of cancer.

Abnormal cytoskeletal structure changes may induce malignant change of normal cells in the body, and affect biological behaviors such as differentiation, proliferation, apoptosis, adhesion, invasion and migration of tumor cells, and even cause drug resistance and chemoradiotherapy resistance of tumor cells^6,7^. Microtubules are one of the components of cytoskeleton. Tubule-promoting polymerins (TPPPs) are a family of proteins that bind to tubulin monomers, promote tubulin polymerization and stabilize the formed microtubules^8^. TPPP3, a member of TPPP family, has been confirmed to be involved in the regulation of these malignant behavior transformation in tumor cells in a number of tumor studies^9–12^. Stabilizing the cytoskeleton structure may effectively inhibit the malignant biological effects and behaviors of glioma cells, and inhibit the proliferation, migration and invasion of tumor cells ^13–15^. Therefore, exploring the influence of TPPP3 on the malignant phenotype of glioblastoma cells can provide a new entry point for us to deal with the refractory of GBM.

Epithelial to mesenchymal transition (EMT), driven by a myriad of transcription factors and repressors, plays a crucial role in the differentiation of multiple tissues and organs, wound healing, tissue remodeling, cancer progression and metastasis^16^. EMT also occurs during non-epithelial cancers, such as GBM progression, refers to a state process with a less epithelial phenotype and a more mesenchymal phenotype. It is characterized by decreased of epithelial markers such as E-cadherin and increased of mesenchymal markers such as N-cadherin and Vimentin; This leads to the weakening of intercellular adhesion and the loss of cell polarity, and finally enhances the ability of tumor invasion and migration^17^. The EMT program is triggered by the activation of core transcription factors, including Snail1/2, ZEB1/2, and Twist1/2^18^. Numerous studies have suggested that these EMT-inducing transcription factors are aberrantly expressed in multiple types of tumors and are known to favor the invasive process^19^.

In this study, we first detected the expression of TPPP3 in various grades of glioma tissues. Then, different glioblastoma cell lines were used to carry out related in vitro cell function experiments and detection of malignant behavior-related proteins to verify the effect of TPPP3 on the proliferation, apoptosis, migration and invasion of glioblastoma cells. In addition, we analyzed the role of TPPP3 in epithelial-mesenchymal transformation by detecting EMT-related marker proteins. The specific mechanism of TPPP3 regulating the malignant progression of glioblastoma was elaborated based on in vitro cytofunctional response experiments, and the correlation between TPPP3 and Snail1 was analyzed. The biological role of TPPP3 in malignant progression of glioblastoma was further clarified by in vivo experiments. Finally, clinical data were used to analyze the effect of TPPP3 on the survival of glioblastoma patients.

## Materials and methods

### Clinical tissues

The glioma samples were taken from patients with glioma in the Department of Neurosurgery, the Affiliated Hospital of Guizhou Medical University. A total of 81 tissues were involved in this study, including 10 normal brain tissues. In Supplementary Table S1 and S2, we summarized the clinicopathological characteristics collected from patients enrolled in this study. All procedures of experiments using human samples were strictly reviewed and approved by the Institute Research Medical Ethics Committee of Guizhou Medical University. All patients enrolled in this study signed consent forms before the initiation of this study.

### Cell culture

Human glioblastoma cell lines (U87MG, U118, A172, LN229, U251) were purchased from the Shanghai Chinese Academy of Sciences Cell Bank. All cells were cultured in DMEM high-sugar medium and FBS (10%), and then placed in a 37 °C, 5% CO_2_ cell incubator. The NHAs human astrocytes were maintained per the manufacturer’s instructions. All experiments were performed in accordance with relevant guidelines and regulations and the study is reported in accordance with ARRIVE guidelines.

### Real-time fluorescence quantitative PCR

Fully lyse cells or tissues with Trizol reagent, RNA extraction kit (AM1931) was used to extract cells and tissues separately according to the instructions. Follow the steps of the RNA Reverse Transcription Kit (Applied Biosystems™, 4368813) to gradually complete the reverse transcription of RNA. The Q-PCR reaction was performed according to the requirements of SYBR Green Master Mix Kit (Applied Biosystems™, 4432346), results were calculated according to the Ct value (2^ΔΔCt^). The primer involved were as follows: β-actin (internal control) primer (forward primer, 5′-CATGTACGTTGCTATCCAGGC-3′; reverse primer, 5′-CTCCTTAATGTCACGCACGAT-3′); TPPP3 primer (forward primer, 5′-GGTCCATTCCTGCGTCGTTC-3′; reverse primer, 5′-GCCCAGTTCTTGCCATTCATC-3. All reagents and kits were purchased from Thermo Scientific.

### Western blot analysis

An appropriate amount of RIPA lysate was used to lysate cells or tissues and extract proteins. Protein concentration was measured with BCA kit (Abcam, ab102536). Protein samples and protein markers were loaded in 10% separation gel and then transferred to 0.45-μm PVDF membranes (Millipore, 42029053) activated with methanol. Then membrane was blocked in 5% non-fat milk for 2 h and incubated with primary antibody at 4 °C overnight. Then placed into the corresponding secondary antibody (ab288151, ab6728, Abcam, USA,1:5000) box and incubated with a shaker at room temperature for 2 h. Finally, the ECL working solution (Thermo Scientific™, USA) was prepared according to 1:1, and the membrane was exposed in the chemiluminescence instrument, the results were analyzed by Image J. The involved primary antibodies were as follow and concentration was diluted according to product instructions: TPPP3 (NBP2-13469, NBP2-95209, Novus Biologicals), and N-cadherin (ab76011, Abcam), E-cadherin (ab40772, Abcam), Vimentin (ab8978, Abcam), Snail1 (13099-1-AP, Proteintech), Slug (ab302780, Abcam), Twist1 (ab175430, Abcam), ZEB1 (ab203829, Abcam), β-catenin(ab203829, Abcam).

### Immunofluorescence

The cells were fixed with 4% paraformaldehyde in the petri dish for 15 min. Then add 0.2%Triton X-100 (Sigma-Aldrich, Germany) 200 μL/dish permeability 10 min. Wash PBS 3 times/2 min, add 200 μL sealing solution/dish at room temperature for 1 h. Dilute the primary antibody with PBS in accordance with the required proportion and incubate at 4 ℃ overnight. The fluorescence secondary antibody (ab7090, ab6728, Abcam, USA) was diluted well, and 200 μL/dish was added, wrapped in tin foil to avoid light, and sealed at room temperature for 2 h. 200 μL/dish of DAPI (ab104139, Abcam, 100 ng/mL) was added and dyed at room temperature for 10 min, with the whole process shielded from light. Finally, photographs are taken under a confocal laser microscope.

### Wound-healing assay

The cells were counted and added with DMEM high glucose medium containing 10% FBS to adjust the cell concentration to 200,000 cells/well. The cells were cultured in cell incubator. When the cells grew to 80–90%, mark “+” at the bottom of the hole with 10 μL spear head, remove the old culture medium, then add 900 μL DMEM serum-free medium containing Mitomycin C (10 μg/mL) to each well, take photos with fluorescence microscope. The cells were photographed again at the same location 24 h later, and their mobility was compared. The closure area of wound was calculated as migration index: migration area (%) = (A0−An)/A0 × 100, where A0 represents the area of initial wound area, An represents the remaining area of wound at the metering point.

### Transewell assay

Add 50 μL Matrigel (Corning, USA) to the upper chamber of each Transwell cell (diluted 1:8 in DMEM serum-free medium) and place in an incubator for 30 min. The cell concentration was adjusted to 100,000 cells/well. Transewell added 600 μL DMEM containing 1% FBS into the 24-well plate in the lower chamber, and 200 μL DMEM medium containing 100,000 cells into the upper chamber (Corning, CLS3470), and cultured in the cell incubator for 24 h. The cells were then placed in a 24-well plate containing 600 μL 4% paraformaldehyde for 30 min and stained with 1% crystal violet (Sigma-Aldrich, V5265) for 30 min. Gently wipe the upper chamber with a cotton swab, dry it, and take photos with a fluorescent microscope for counting.

### Cell count kit-8(CCK8) assay

When the cells grew to 70–80% and fused, the adherent cells were digested with trypsin. the adherent cells were collected and counted to prepare cell suspension with a density of 5 × 104/mL, and 100 μL cell suspension was prepared in a 96-well plate. The culture plate was pre-cultured in the incubator for 24 h. According to the instruction of CCK8 kit (Dojindo, Japan), 10 μl working solution was added to each well. After incubation for one hour, the absorbance at 450 nm was measured with a microplate reader.

### Flow cytometry analysis

The apoptosis rate of GBM cells was analyzed with propidium iodide (PI)/Annexin V Cell Apoptosis Kit (Invitrogen, V13245) in reference to manufacturer’s suggestions. In short, after 48 h transfection, GBM cells were stained by FITC-Annexin V and PI. Finally, cell apoptosis rate was examined by flow cytometry (FACSCantoII, 338960; BD Biosciences, San Jose, CA, USA). The experiment was repeated for three times.

### Immunohistochemistry

The prepared paraffin sections were stained by Immunohistochemistry. The staining was completed according to the description of the immunohistochemical staining kit (CST, SignalStain® Boost) instructions. The brief steps are as follows: the dewaxed tissue sections were placed in anhydrous ethanol for 15 min, 95%, 90%, 80% and 75% ethanol for 10 min successively, and finally placed in ddH_2_O for 15 min for dehydration. The sections were immersed in 1× citrate repair solution, then heated in the microwave oven until boiling, and kept at temperature (95°–98°) for 10 min. Then the sections were cooled for 30 min for antigen repair. After inactivation of endogenous peroxidase in tissues, the slices were closed and incubated in diluted primary and secondary antibodies. DAB chromogenic solution was added on the surface of the slices, and the reaction lasted until brownish yellow color appeared. The chromogenic solution was removed, and the slices were cleaned with PBS, then hematoxylin was added for redyeing for 3 min. Finally, it is observed and photographed under a microscope.

The stained sections were observed by two pathologists under a 200× microscope and evaluated independently. The immune response score was calculated by the following formula, IS = percentage (0, no target molecule expressed; 1, 1–10% was positive; 2, 11–50% is positive; 3, > 50% is positive) x staining intensity (0, no color; 1, weak color rendering; 2, medium color display; 3, strong color). When the IS score is between 3 and 9, it means that the target protein is highly expressed, otherwise the target protein is considered to be low-expressed.

### Transfection

Cell lines were constructed using lentivirus transduction as described previously^20^. Lentivirus (Genechem, China) was packaged in 293T cells and transfected with Lipofectamine 3000 liposome transfection reagent (Thermo Scientific, L3000001). The interference sequence of TPPP3 as sh1:5′-CCGGGCCAATGTGGGCGTCACTAAACTCGAGTTTAGTGACGCCCACATTGGCTTTTTG-3′, sh2:5′-CCGGCTGCTCGGGTCATCAACTATGCTCGAGCATAGTTGATGACCCGAGCAGTTTTTG-3′, sh3:5′-CCGGAGGAGAGCTTCCGCAAGTTTGCTCGAGCAAACTTGCGGAAGCTCTCCTTTTTTG-3. Lentiviral constructs stabilized expressing TPPP 3 or Snail1 were obtained from Addgene. When the cells in the blank control die and the lentivirus-transfected cells are still alive, the selected cells are expanded and sub-cultured, and cells were collected to detect the expression of the target protein.

### Xenograft mouse model

Animal experiments are approved by the Ethics committee and carried out in strict accordance with the requirements. Twenty-four 5-week-old female BALB/c-nude mice were selected from Shanghai experimental animal center of Chinese Academy of Sciences for xenotransplantation experiment. U251 cells (shCrtl, shTPPP3) with a cell density of 5 × 10^7^/mL were injected into the right flank of mice. The size of the tumor was measured with a vernier caliper every 5 days after tumor formation, the tumor volume = (width^2^ × length) / 2, the curve was plotted based on time–weight. After 30 days of subcutaneous inoculation, the nude mice were sacrificed by neck-method, and the tumor tissues in vivo were separated for weighing, photographing, and subsequent experiments. All animal experiments were conducted according to the approved protocol by the Animal Welfare Ethical Review Committee.

### Statistics analysis

In this study, GraphPad Prism software was used for statistical analysis of data. Student's test was used for significant differences among different groups. Kaplan–Meier survival curve and log-rank test were used to analyze survival differences. A *P* value less than 0.05 was considered statistically significant.

### Ethics approval and consent to participate

The study was approved by the Ethics Committee of the Affiliated Hospital of Guizhou Medical University. Signed written informed consents were obtained from the patients and/or guardians.

## Results

### The expression of TPPP3 in glioma is higher than that in normal brain tissue (NBT)

**Figure 1:**
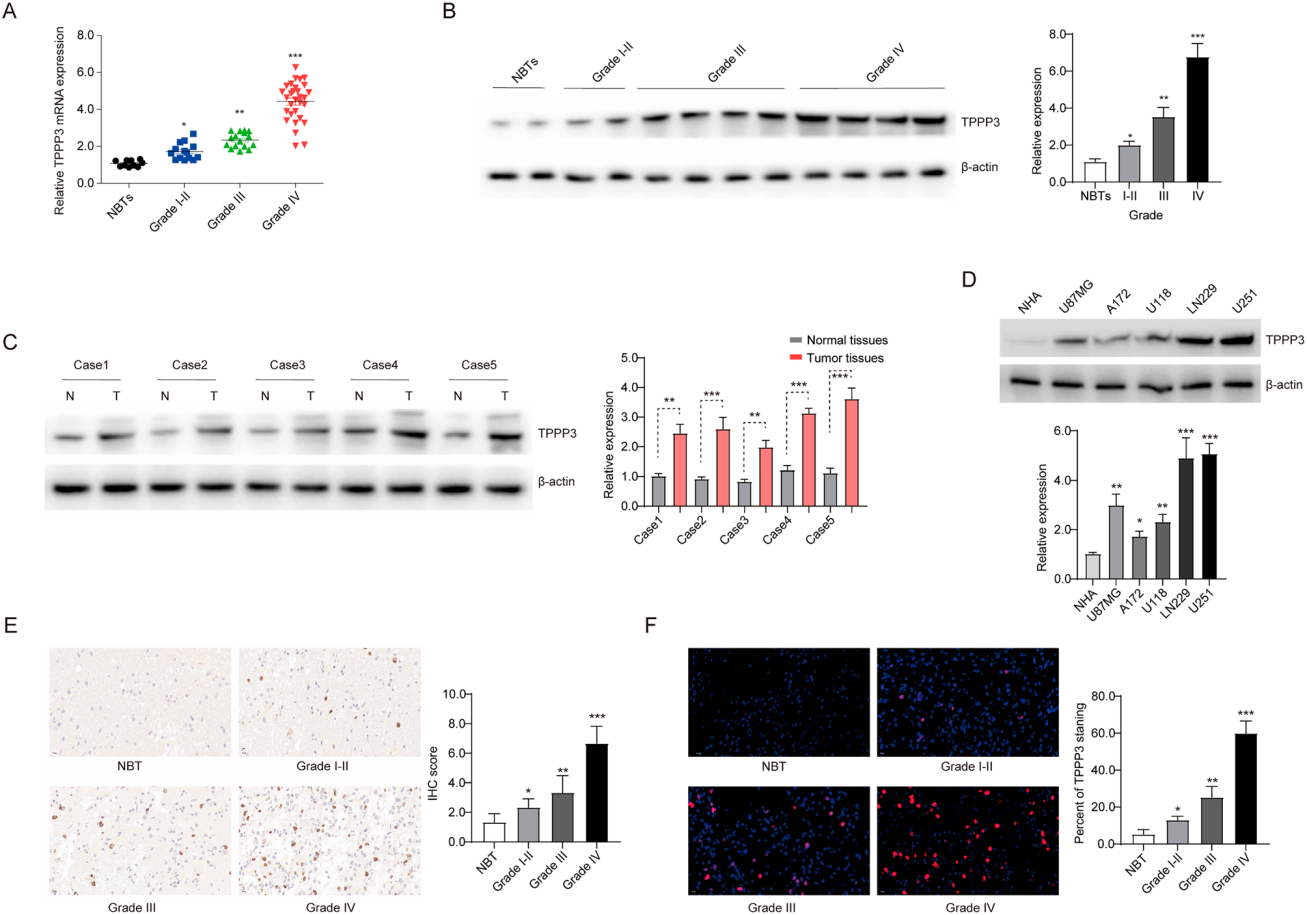
TPPP3 was highly expressed in glioma tissues and glioma cells, and increased with the increase of glioma grade. (**A**) The expression of TPPP3 in different grades of glioma and brain tissue at the mRNA level was detected by qRT-PCR assay. (**B**) Western blot detection the expression of TPPP3 in clinical glioma specimens of various grades. The right panel is quantification results. (**C**) The expression of TPPP3 in 5 glioblastoma tissues and normal tissues was analyzed by Western Bolt. The right panel is quantification results. (**D**) The level of protein expression of TPPP3 in each line of glioblastoma cells. n = 3 each group. The lower panel is quantification results. (**E**, **F**) The expression of TPPP3 in different grades of glioma tissues was independently confirmed by immunohistochemistry and immunofluorescence. n = 6 each group. The right panel is quantification results of immunohistochemistry and immunofluorescence assay. Data from at least three independent experiments were quantified. *P < 0.05, **P < 0.01, ***P < 0.001.

We performed surgical separation of glioma and normal brain tissues from 81 patients, and used real-time fluorescence quantitative PCR to quantitatively analyze the mRNA abundance of TPPP3 in randomly selected surgically separated tissues and normal brain tissues. It was found that the expression of TPPP3 in gliomas was higher than that in normal brain tissue (NBT), and it became more pronounced as the grade of gliomas increased (Fig. 1A). In terms of protein expression level, we used Western blot analysis to detect the expression level of NBT and various grades of glioma tissues, and the conclusion is the same as above (Fig. 1B). We randomly selected 5 pairs of glioblastoma and normal brain tissues for Western blot experiments, and the results suggested that TPPP3 was more highly expressed in tumor tissues (Fig. 1C). As shown in Fig. 1D, TPPP3 expression was found higher in glioblastoma cell lines than in human normal astrocytes (NHA). IHC and IF were performed to further confirm the expression of TPPP3 in different grades of glioma tissue and normal brain tissue. The results presented are consistent with the above results (Fig. 1E, F).

### TPPP3 affected the epithelia-mesenchymal transition (EMT) process of glioblastoma

**Figure 2:**
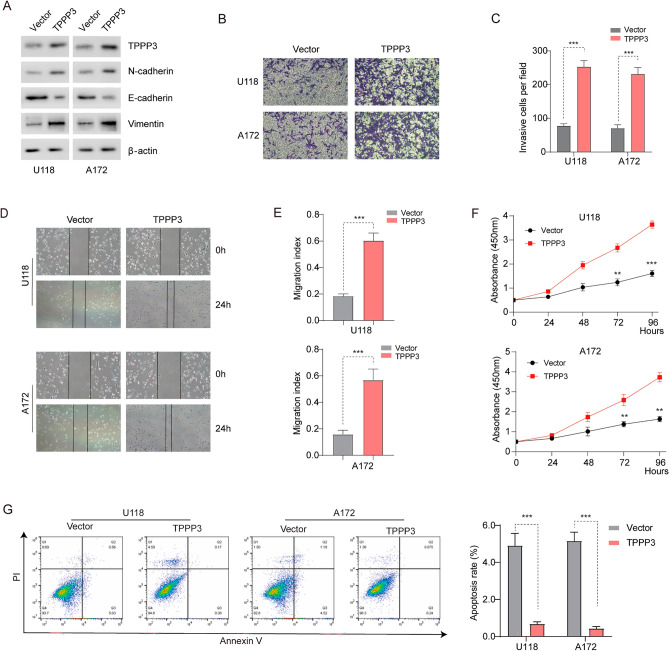
TPPP3 was associated with the EMT process of glioblastoma cells and promoted the proliferation, migration and invasion of glioblastoma cells. (**A**) Western blot detection the effect of TPPP3 overexpression on the expression of EMT pathway related proteins. (**B**, **C**) The effect of TPPP3 overexpression on glioblastoma cells invasion was analyzed by Transwell assay. n = 4 each group. (**D**, **E**) Wound healing assay quantified the effect of TPPP3 overexpression on glioblastoma cell migration. n = 5 each group. (**F**) CCK8 assay detected the proliferation of glioblastoma cells after overexpression of TPPP3. (**G**) Flow cytometry analysis detected the apoptosis in U118 and A172 cells after TPPP3 overexpression. n = 4 each group. Data from at least three independent experiments were quantified. **P < 0.01, ***P < 0.001.

In order to verify the biological function of TPPP3 on glioblastoma, we selected existing glioblastoma cell lines in the experiment for analysis. The results suggested that after overexpression of TPPP3, the related proteins of the EMT pathway changed (Fig. 2A). We conducted a functional experiment to test the proliferation ability of glioblastoma cells. Transwell results suggested that the number of cells that crossed the compartment and penetrated the matrix glue and invaded the opposite side of the compartment cellulose membrane increased significantly after TPPP3 was overexpressed (Fig. 2B, C). Wound healing experiments suggested similar results: When TPPP3 expression was increased, the migration ability of glioblastoma cells was significantly enhanced compared with the control group (Fig. 2D, E). CCK8 results indicated that the proliferation rate of glioblastoma cells after TPPP3 overexpression was significantly higher than control group (Fig. 2F). Furthermore, flow cytometry analysis showed that overexpressing TPPP3 significantly inhibited the apoptosis compared to the control cells (Fig. 2G). These data suggest that up-regulation of TPPP3 expression in glioblastoma cells may confer stronger ability of migration, invasion, proliferation and lower apoptosis in vitro.

### TPPP3 expression silencing inhibited the malignant biological behavior of glioblastoma cells

**Figure 3:**
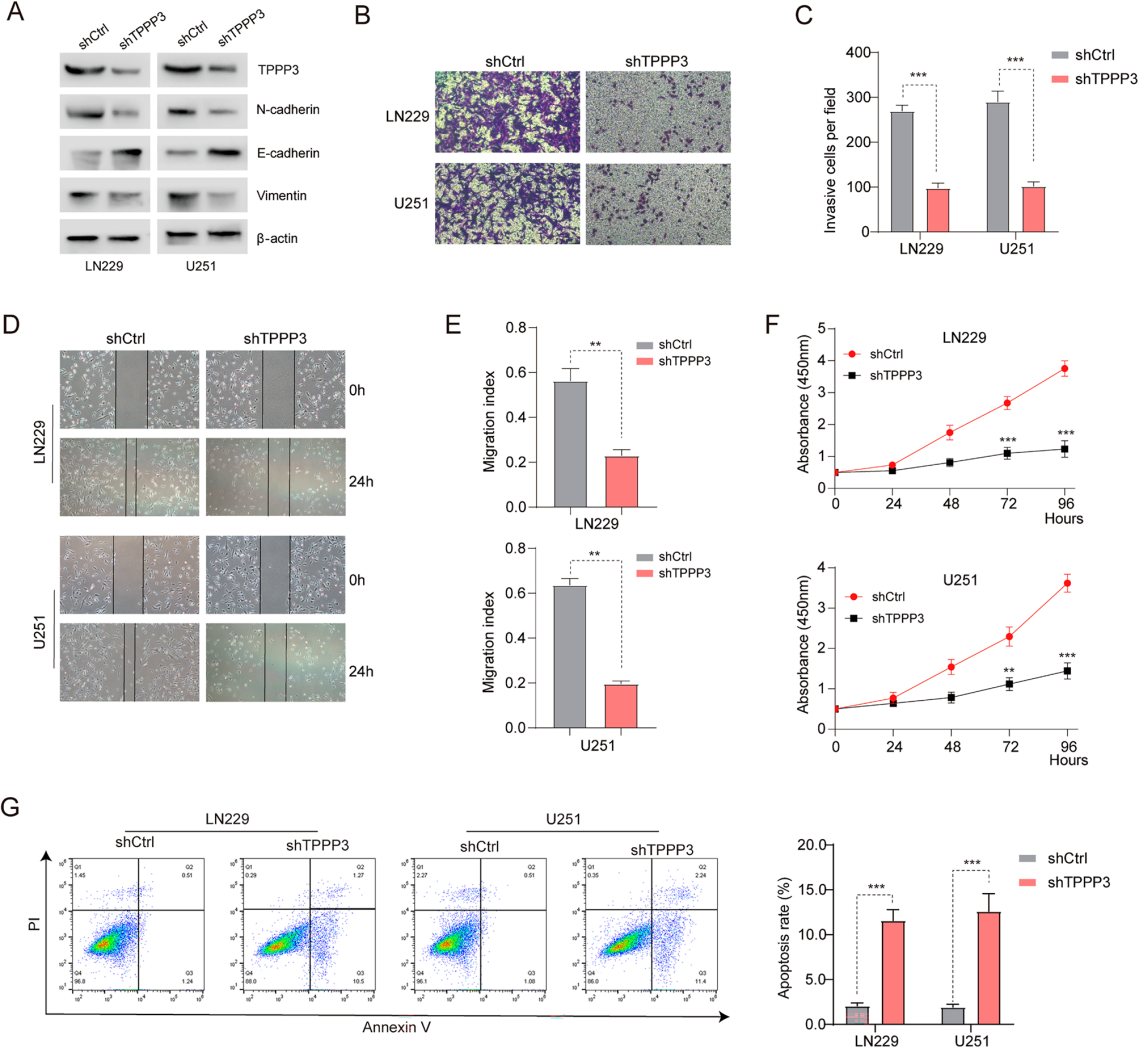
Knockdown of TPPP3 expression inhibited the EMT process and malignant biological behavior of glioblastoma cells. Glioblastoma cell lines with TPPP3 knockdown expression were constructed for subsequent experiments. (**A**) Changes in the expression of key proteins in the EMT process of glioblastoma cells after knocking down the expression of TPPP3. (**B**, **C**) The invasion ability of glioblastoma cells was quantitatively analyzed by Transwell experiment. n = 4 each group. (**D**, **E**) The number of migrated glioblastoma cells was quantitatively analyzed by the wound-healing assay. n = 4 each group. (**F**) CCK8 assay detection the proliferation of glioblastoma cells. Data from at least three independent experiments were quantified. (**G**) Flow cytometry analysis detected the apoptosis in LN229 and U251 cells after TPPP3 silence. n = 4 each group. **P < 0.01, ***P < 0.001.

To further explore the mechanistic role of TPPP3 in regulating glioblastoma cell invasion and migration, we knocked down TPPP3 in LN229 and U251 cells (Fig. 3A). TPPP3 silencing significantly decreased N-cadherin and Vimentin levels, and increased that of E-cadherin (Fig. 3A). The effect of TPPP3 knockdown on the migration and invasion of glioblastoma cells was evaluated by wound healing assay and Transwell assay. Transwell assay showed that the migration number of LN229-shTPPP3 cells was significantly lower than that of maternal cells (Fig. 3B, C). Wound healing experiment results showed that the migration rate of LN229-shTPPP3 was significantly lower than that of maternal cells (Fig. 3D, E). Similarly, Transwell migration experiment and wound healing experiment were performed in U251 and U251-shTPPP3 cells, and similar results were obtained. In addition, 96 h CCK-8 assay showed that the proliferation ability of glioblastoma cells with TPPP3 knockdown was significantly reduced compared with control cells (Fig. 3F). The flow cytometry analysis (Fig. 3G) further confirmed that TPPP3 knockdown significantly induced the apoptosis of the GBM cells. These results indicated that TPPP3 knockdown could inhibit the proliferation, migration, invasion and induce apoptosis of glioblastoma cells.

### TPPP3 affected the process of EMT through the key protein Snail1

**Figure 4:**
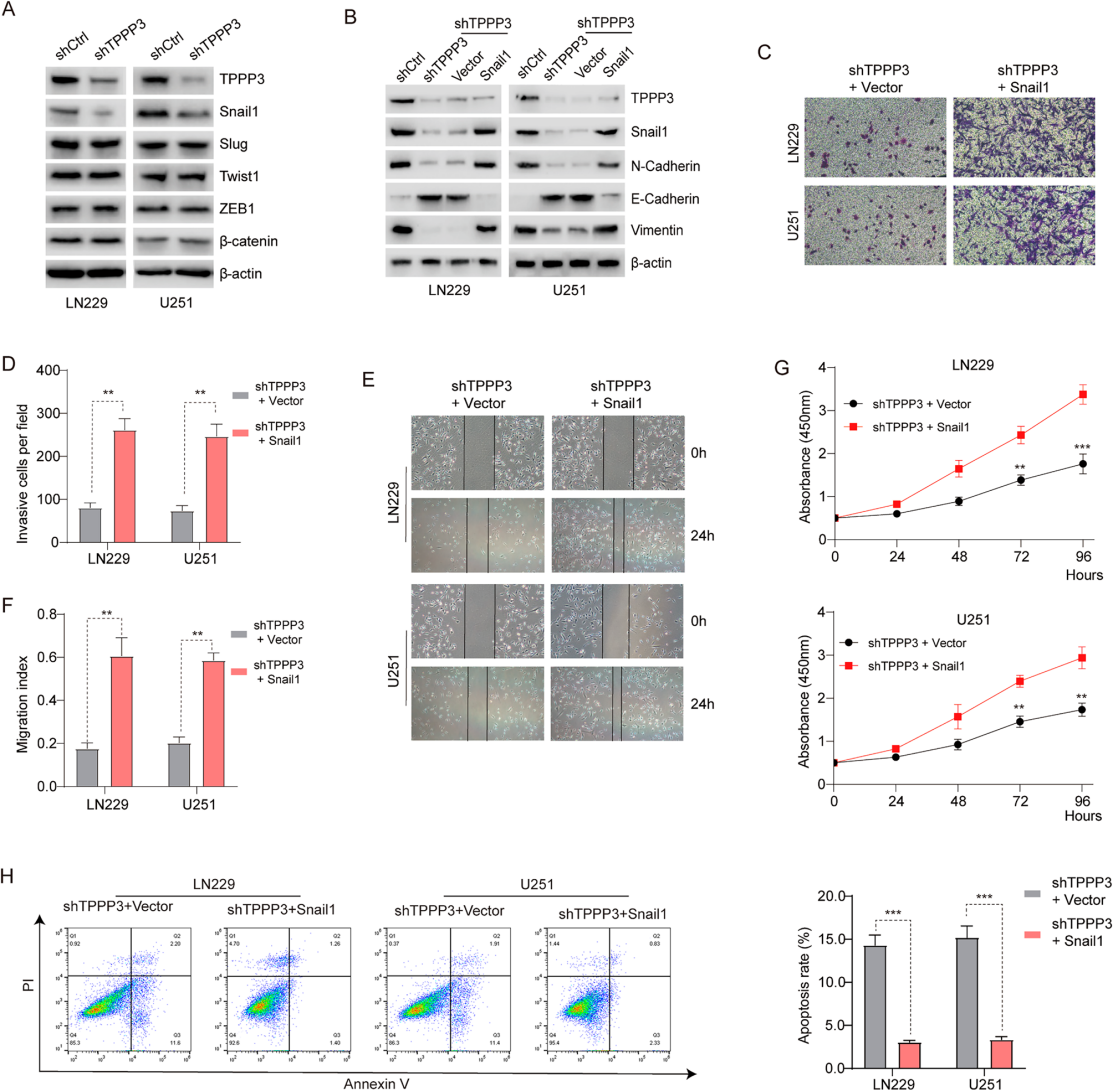
TPPP3 induced epithelial-mesenchymal transition by regulating the expression of Snail1 protein. (**A**) Western Blot analysis the effect of knocking down the expression of TPPP3 on the expression of common key proteins in the EMT process. (**B**) The effect of overexpression of Snail1 in cells with knockdown of TPPP3 on the expression of E-cadherin, N-cadherin and Vimentin, the key markers of EMT process. (**C**, **D**) Snail1 was overexpressed in TPPP3 knockdown cells, and the invasion ability of glioma cells was quantitatively analyzed by Transwell experiment. n = 5 each group. (**E**, **F**) The wound healing experiment tested the migration ability of glioblastoma cells after overexpression of Snail1 in TPPP3 knockdown cells. n = 5 each group. (**G**) The CCK8 experiment analyzed the effect of overexpression of Snail1 in TPPP3 knockdown cells on the proliferation of glioblastoma cells. (**H**) Flow cytometry analysis detected the apoptosis of glioblastoma cells after overexpression of Snail1 in TPPP3 knockdown cells. n = 4 each group. Data from at least three independent experiments were quantified. **P < 0.01, ***P < 0.001.

The previous data analysis results showed that TPPP3 expression increased with the increase of the degree of malignancy and could target the relatively malignant mesenchymal phenotype, while the transformation of epithelioid cells to mesenchymal phenotype in tumor cells quietly occurred in the progression of tumor malignancy, and this transformation is also the source of tumor invasion and metastasis ability. In LN229 and U251 cell models, the expression levels of common key markers of EMT process in TPPP3 interference group and corresponding control group were detected by Western blot analysis. The results indicated that the down-regulation of TPPP3 expression level could inhibit Snail1 expression, while the expression of other key proteins had no significant effect (Fig. 4A). In order to further verify whether Snail1 is a key protein for TPPP3 to play a role in the EMT process, we conducted a rescue experiment. The results suggested that the down-regulation of TPPP3 expression level could inhibit the expression of N-cadherin and Vimentin, while the expression of E-cadherin protein was negatively correlated with the expression of TPPP3. On the basis of low expression of TPPP3, overexpression of Snail1 protein, the experimental results had changed. That is, the effect of down-regulation of TPPP3 on the expression of N-cadherin and Vimentin was negligible. When Snail1 was overexpressed and TPPP3 was at a low expression level, the negative correlation between E-cadherin protein expression and TPPP3 expression no longer existed (Fig. 4B).

In functional experiments, similar results were also found. Transwell experiments suggested that in glioblastoma cell lines with low expression of TPPP3, the invasive ability of shTPPP3 glioblastoma cell lines had changed from weak to stronger if Snail1 protein was overexpressed (Fig. 4C, D). Wound-healing assay results showed that the migration ability of shTPPP3 glioblastoma cell line was significantly enhanced compared with the control group after overexpression of Snail1 protein (Fig. 4E, F). In terms of cell proliferation ability, after overexpression of Snail1 protein, the proliferation rate of shTPPP3 glioblastoma cells was significantly faster than that of control cells (Fig. 4G). In addition, knocking down TPPP3 increased apoptotic rate of GBM cells could be largely abolished after Snail1 up-regulation (Fig. 4H).

### The effect of TPPP3 on the proliferation of glioblastoma cells in vivo

**Figure 5:**
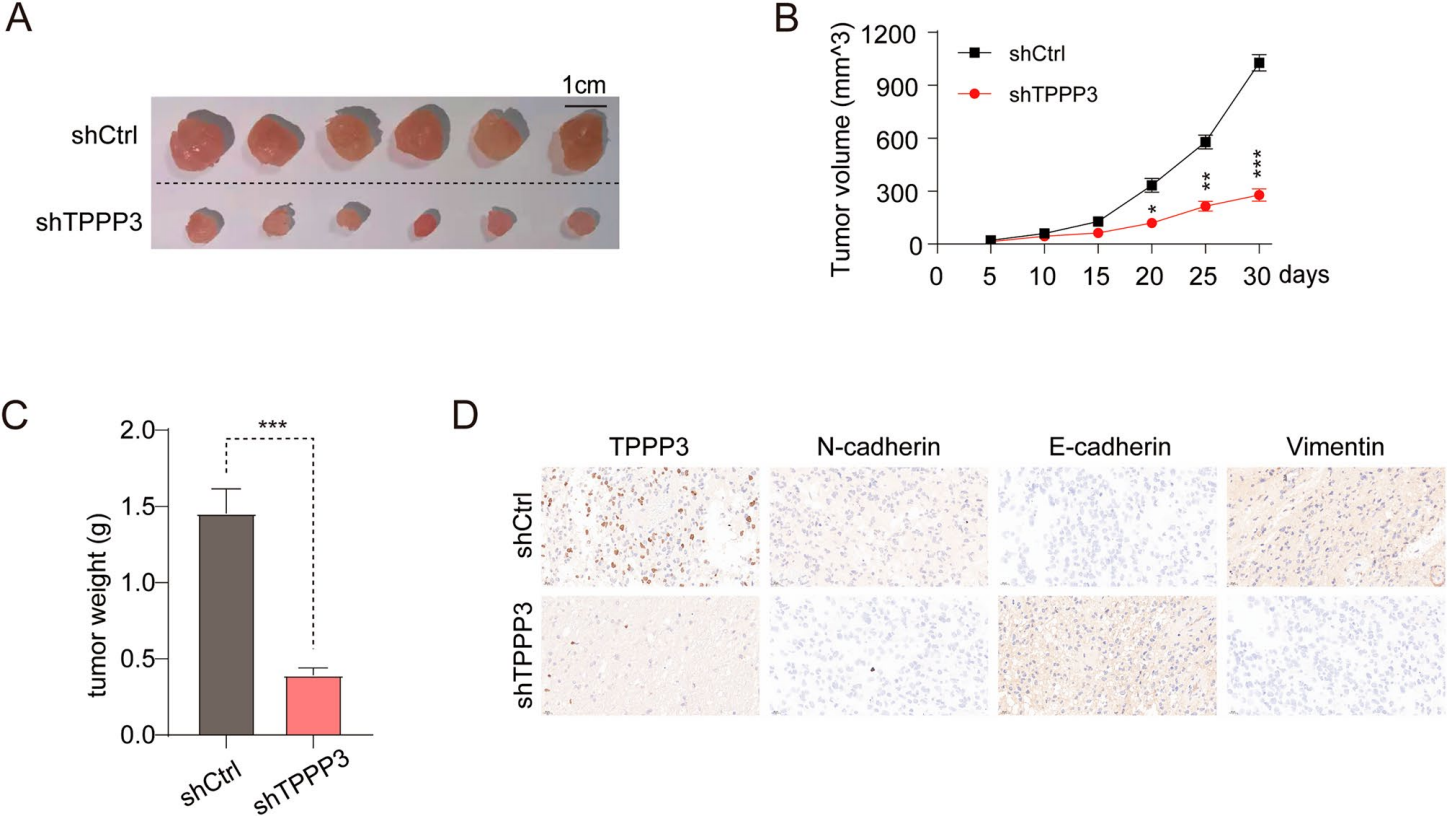
Knockdown of TPPP3 expression significantly inhibited the proliferation of glioblastoma cell lines in vivo. An animal model with TPPP3 knockdown of U251 cells was constructed for subsequent experiments. (**A**) Photos of subcutaneous tumors of mice in the experimental group and the control group. (**B**) Tumor volume of experimental and control mice at different time. (**C**) Tumor weight of experimental and control mice. (**D**) The expression of key proteins in EMT process in tumor tissues of the two groups was detected by immunohistochemistry.

In order to more accurately describe the function of TPPP3 in the occurrence and malignant development of glioblastoma, we knocked down TPPP3 in U251 cells and verified the function of TPPP3 in vivo. It can be seen from Fig. 5A–C that the U251 cells knocked down TPPP3 had the worst tumorigenesis effect in nude mice, and the tumor volume and weight were significantly smaller than the control group. The results of immunohistochemistry suggested that the expression levels of key marker proteins N-cadherin and Vimentin in the EMT process in mouse tumor tissues with knockdown of TPPP3 expression were low, while the expression of E-cadherin protein was negatively correlated with the expression of TPPP3 (Fig. 5D). These in vivo experimental data further illustrate that TPPP3 inhibited the proliferation of glioblastoma cells and affected the EMT process.

### Analysis of TPPP3 and Snail1 protein expression in clinical samples of patients with glioblastoma

**Figure 6:**
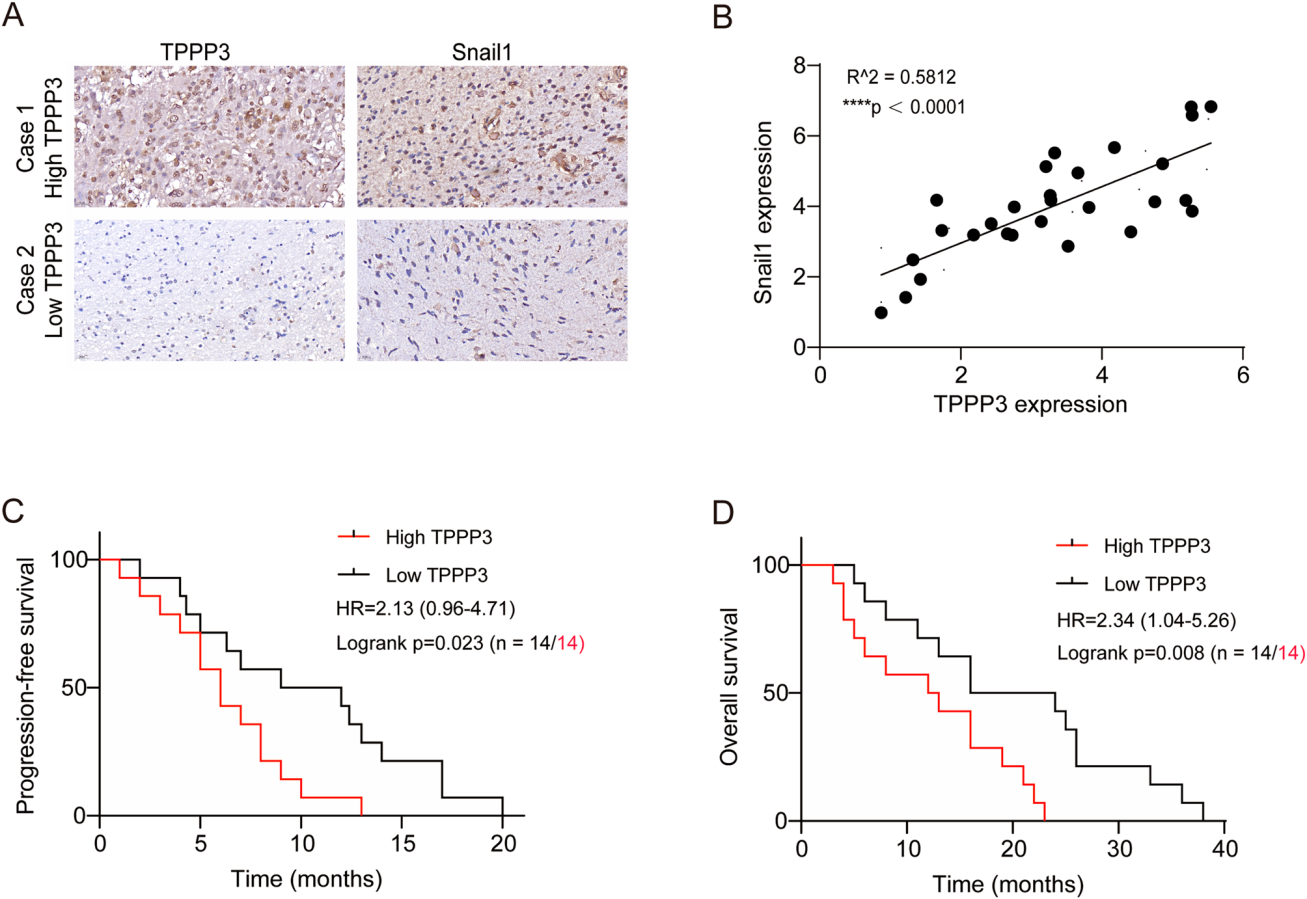
Data analysis of clinical significance of TPPP3 in glioblastoma. (**A**) Immunohistochemical images of representative glioblastoma tissues with high and low TPPP3 expression. (**B**) Correlation analysis between TPPP3 and Snail1 in glioblastoma tissues. (**C**, **D**) The expression level of TPPP3 in glioblastoma affected the survival of patients. HR, Hazard Ratio. ****P < 0.0001.

We randomly selected some glioblastoma tissue with high and low expression of TPPP3. The results of immunohistochemistry suggest that the expression of Snail1 changes with the expression of TPPP3 (Fig. 6A). There was a positive correlation between the expression of Snail1 and the expression of TPPP3 (Fig. 6B). At the same time, we conducted survival analysis on the existing data and found that among glioblastoma patients, patients with low TPPP3 expression had a longer progression-free survival (Fig. 6C), and the overall survival rate was higher than that of the high TPPP3 group (Fig. 6D). These results were basically consistent with the above experimental results.

## Discussion

Glioma is the tumor type with the highest rate of intracranial disability and mortality. According to the histopathological characteristics of glioma, the World Health Organization classified glioma into grade I–IV, which represents the degree of malignancy. Grade I–II is low-grade glioma (LGG), and grade III–IV is high-grade glioma (HGG)^21^. The invasion of glioblastoma cells into regions of the normal brain tissue is a major cause for the poor prognosis of malignant glioblastomas, but the underlying mechanisms governing tissue invasion and metastasis remain incompletely understood. our study found relevant mechanisms of TPPP3 regulating tumor cell migration, invasion, proliferation, apoptosis and EMT transformation in glioblastoma, and discussed the correlation and association between TPPP3 and EMT marker protein Snail1 in detail. This study will pave the way for further exploration of the role of TPPP3 in glioblastoma, provide direction and ideas, and provide theoretical evidence for TPPP3 to become a potential therapeutic target for glioblastoma.

While our results revealed that TPPP3 played a key role in glioblastoma EMT, the deeper mechanism remains unclear. The actin cytoskeleton is a molecular framework that provides physical support for cell structure and proliferation^14^. Studies reported the relationship between the cytoskeleton and glioma, that was, AVIL, a regulator of the cytoskeleton, could drive the tumorigenesis of glioblastoma^22^. TPPP3 protein can promote microtubule polymerization^23^. Studies have found that interfering TPPP3 expression can inhibit Hela cell colonization, block G1/S phase, and promote cell apoptosis^24^. In addition, in nude mouse models, studies have found that Lewis Lung Carcinoma cells that inhibit TPPP3 expression formed subcutaneous tumors in mice smaller and slower than the control^25^. Shukla et al.^26^ found that TPPP3 plays an important role in the demulsification process, and inhibition of TPPP3 can induce mitochondria-dependent apoptosis. These results suggest that inhibition of TPPP3 expression can interfere with the process of mitosis and cell cycle and thus inhibit the proliferation of tumor cells. The role of TPPP3 in some malignancies is also gradually being discovered. Yang et al., believed that TPPP3 involves a variety of immune-related pathways and wad related to the level of immune infiltration. And TPPP3 could be considered as a biomarker for predicting the prognosis and immune invasion of head and neck squamous cell carcinoma^10^. TPPP3 plays different roles in different tumors. Inhibit the proliferation, invasion and migration of cervical endometrial carcinoma^11^, and nasopharyngeal carcinoma^12^. by different ways. But in breast cancer^27^ and non-small cell lung cancer^9^, there was a carcinogenic effect. Our research found that TPPP3 was an oncogene in glioma cells, which promoted the malignant biological behavior of glioblastoma cells.

Epithelial-mesenchymal transition (EMT) is the source that enables tumor cells to acquire strong invasion ability and thus promote tumor progression and metastasis^28^. In cancer, EMT is associated with tumor initiation, invasion, metastasis, proliferation and resistance to therapy^16^. EMT is associated with the proliferation, migration and invasion of glioma cells^29^, the role of EMT in the development and progression of glioblastoma is a hot research topic^30^. It was reported that the new functional glioma stem cell marker LGR5 played a role in glioma by promoting EMT^31^. Xie et al.^32^ found that Eukaryotic translation Factor 1δ promotes proliferation, migration and invasion of glioma cells through EMT. It had also been reported that IDH1 mutation promoted glioblastoma cell proliferation and migration by inducing EMT^33^. Some scientists have elaborated on the role of EMT in the prognostic value of glioma patients and whether it plays a role in the immune microenvironment of glioma^34^. To assess whether the effect of TPPP3 on glioblastoma motility is related to EMT, we examined the expression of EMT-related markers (Vimentin, N-cadherin, e-cadherin). The results showed that Vimentin and N-cadherin expression were down-regulated after TPPP3 expression was decreased, while E-cadherin expression was up-regulated. This finding suggests that the regulation of glioblastoma motility by TPPP3 may be related to epithelial mesenchymal transformation. The mesenchymal phenotype is the most aggressive, malignant, and proliferative class of glioblastoma. Snail1, a member of the SNAIL family of transcriptional repressors and a key EMT regulator, is aberrantly expressed in various cancers where it is posited to regulate diverse processes ranging from tumor cell invasion, proliferation and metastasis^35^. Up-regulation of TPPP3 expression in glioblastoma cells may confer stronger ability of migration, invasion, proliferation and lower apoptosis in vitro. TPPP3 knockdown could inhibit the proliferation, migration and, invasion and induce apoptosis of glioblastoma cells, that could be largely reversed after Snail1 up-regulation. Our data indicated that TPPP3 may promote the migration, invasion, proliferation and inhibit apoptosis of glioma cells via Snail1 by accelerating EMT. In addition, we verified that only Snail1 levels were significantly altered in GBM cell lines with knockdown or overexpression of TPPP3, whereas the other EMT-related transcription factors were unaffected. As such, we hypothesized that TPPP3 regulates EMT in the GBM cells by activating Snail1. This further clarified the biological role of TPPP3 in the malignant progression of glioblastoma. We hypothesized that TPPP3 is functionally coupled with and capable of modulating Snail1 function. The Snail family proteins are also labile proteins, and can be rapidly degraded by the proteasome system. It has also been reported that RND3 promotes Snail1 degradation and inhibits migration and invasion of glioblastoma cells, which also confirms Snail1’s role in glioma cells^36^. Another report confirmed the role of Snail1 in the process of MicroRNA-22 regulating the proliferation, drug sensitivity and metastasis of human glioma cells^37^. Therefore, whether TPPP3 regulates the stability of Snial1 at the transcriptional level or through post-translational modification remains to be explored. These findings suggested the role of Snail1 in EMT and glioma, which made our findings more credible.

After the analysis of clinical data, we learned that there was a positive correlation between TPPP3 and Snail1, and the role of TPPP3 in the survival of glioblastoma patients was also presented. Our results suggest that TPPP3 may have a role in glioblastoma. However, we still need more evidence to support the application value of TPPP3 in glioblastoma, and the detailed mechanism of its participation in the malignant biological behavior of glioblastoma needs to be explored.

## Conclusions

In conclusion, TPPP3 expression significantly increased in glioma tissues and cells, and interfering with TPPP3 expression significantly inhibited the proliferation, migration and invasion of glioblastoma cells, and the mechanism acts affected the process of EMT by regulating the expression of Snail1 protein. The content of this study paves the way for further in-depth exploration of the role of TPPP3 in glioblastoma in the future, and provides new treatment and research directions.

### Supplementary Information

Supplementary Tables.

Supplementary Figures.

## Data Availability

The datasets used and/or analyzed during the current study are available from the corresponding author on reasonable request. Unprocessed images of Western blot were added as Supplementary Figures.
