# Supplementary material for: TPPP3 promote epithelial-mesenchymal transition via Snail1 in glioblastoma
Source: Sci Rep. 2023 Oct 20;13:17960. doi: 10.1038/s41598-023-45233-w (PMC10589222; doi:10.1038/s41598-023-45233-w)
Supplement: Supplementary file 1 — Supplementary Tables. [file 41598_2023_45233_MOESM1_ESM.docx]

**Supplementary Table S1. 28 GBM patients clinicopathological characteristic**


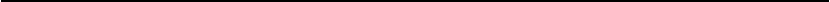


Characteristic Value


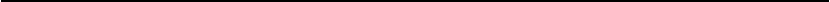


| Total samples (n) | 28 |
| --- | --- |
| Sex (n) |  |
| Male | 13 |
| Female | 15 |
| Medium age, years (range) | 50 (22-73) |
| Tumor location |  |
| Frontal | 15 |
| Non-frontal | 13 |
| Medium KPS (range) | 80 (40-90) |


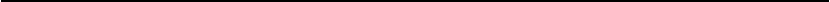


Abbreviations: KPS, Karnofsky performance status; WHO, World Health Organization.

**Supplementary Table S2. 71 glioma patients clinicopathological characteristic**


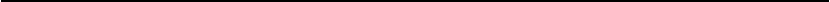


Characteristic Value


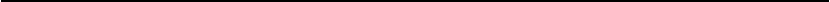


| Total samples (n) | 71 |
| --- | --- |
| Sex (n) |  |
| Male | 37 |
| Female | 34 |
| Medium age, years (range) | 52 (24-77) |
| Tumor location |  |
| Frontal | 39 |
| Non-frontal | 32 |
| Medium KPS (range) | 70 (40-90) |
| WHO grade |  |
| grade I | 8 |
| grade II | 13 |
| grade III | 22 |
| grade IV | 28 |


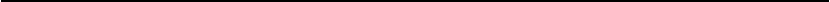


Abbreviations: KPS, Karnofsky performance status; WHO, World Health Organization.
