# Supplementary figures and images for: TPPP3 promote epithelial-mesenchymal transition via Snail1 in glioblastoma
Source: Sci Rep. 2023 Oct 20;13:17960. doi: 10.1038/s41598-023-45233-w (PMC10589222; doi:10.1038/s41598-023-45233-w)

**Figure 1B**

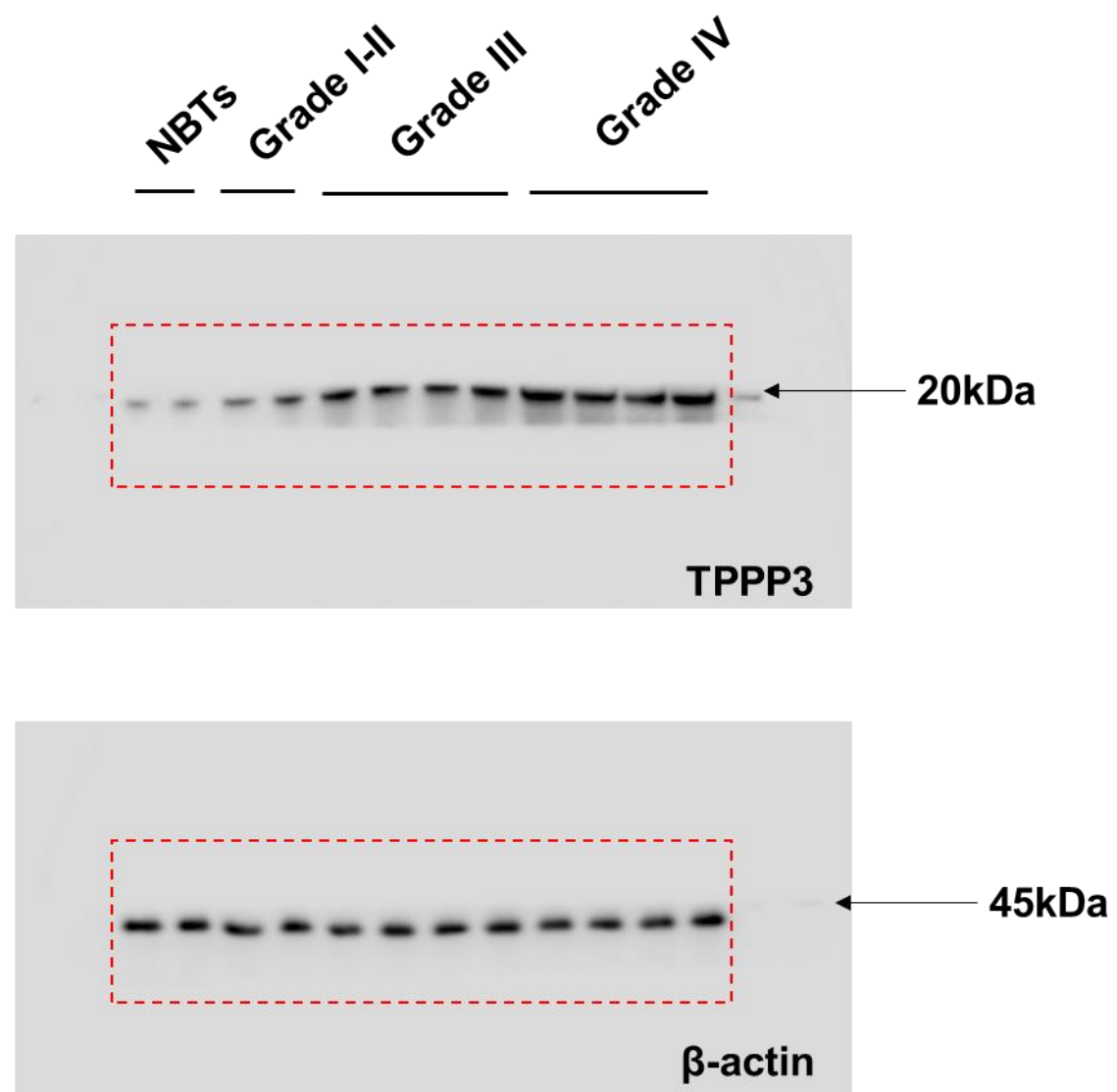

Figure 1C

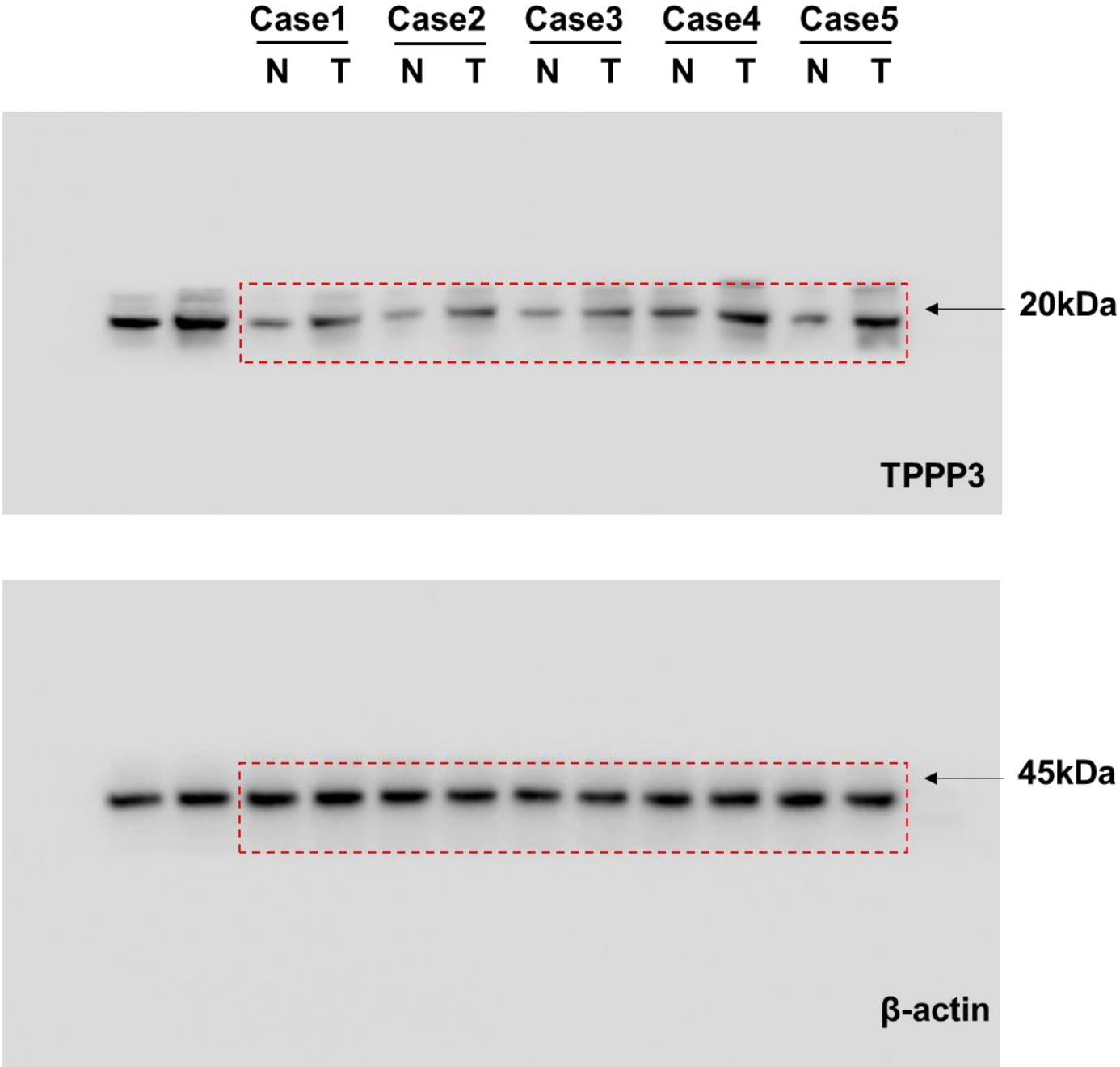

**Figure 1D**

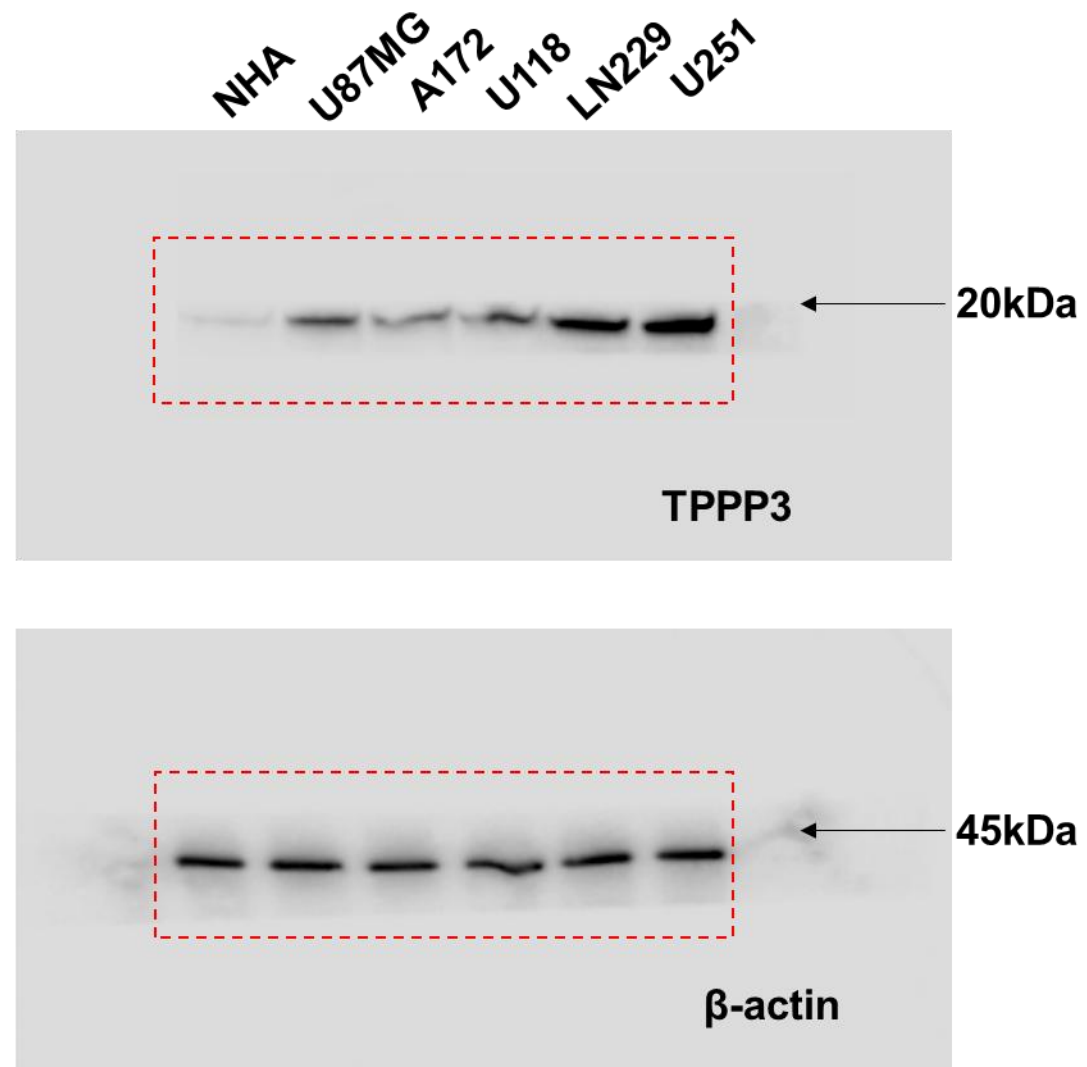

**Figure 2A**

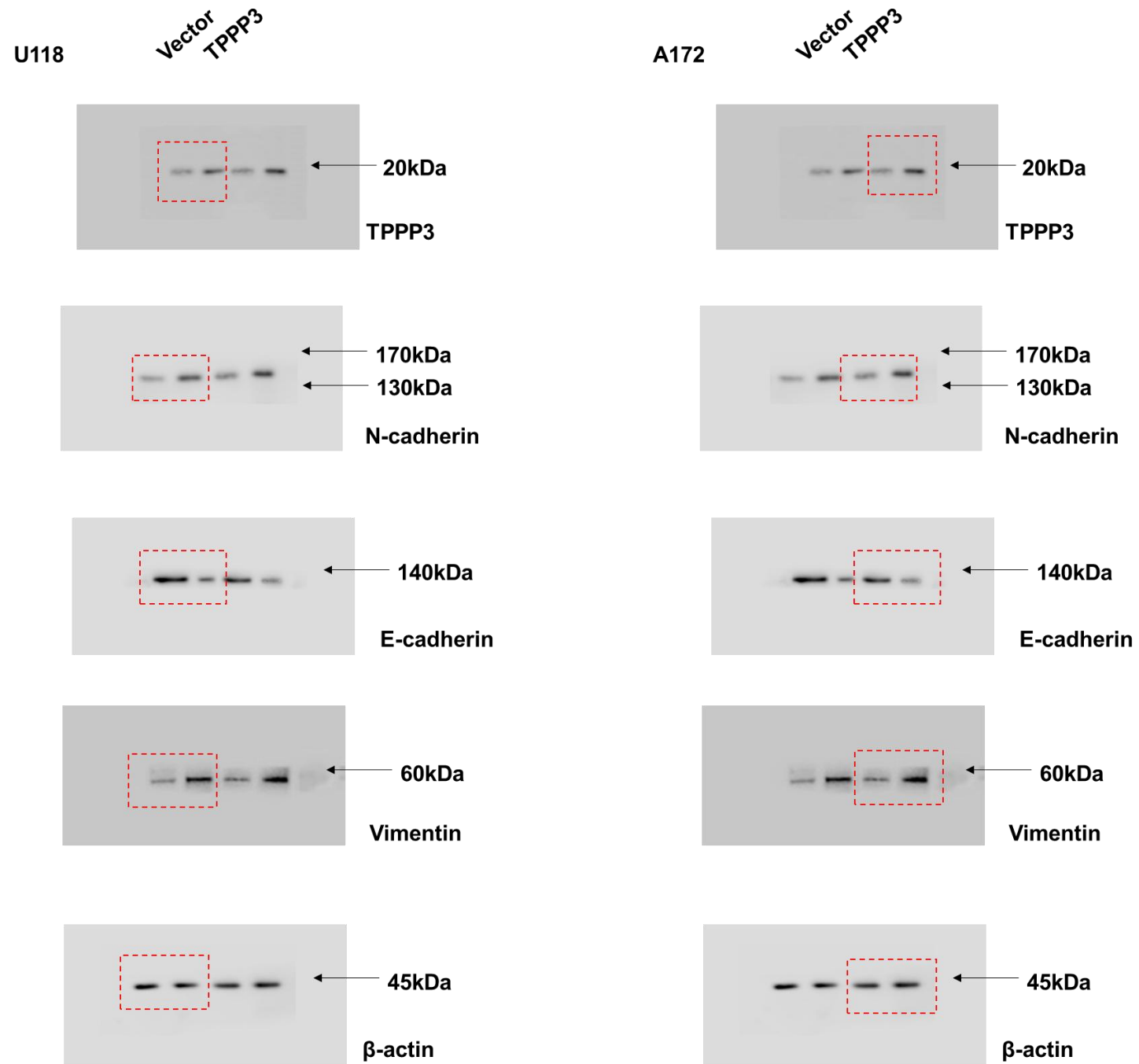

**Figure 3A**

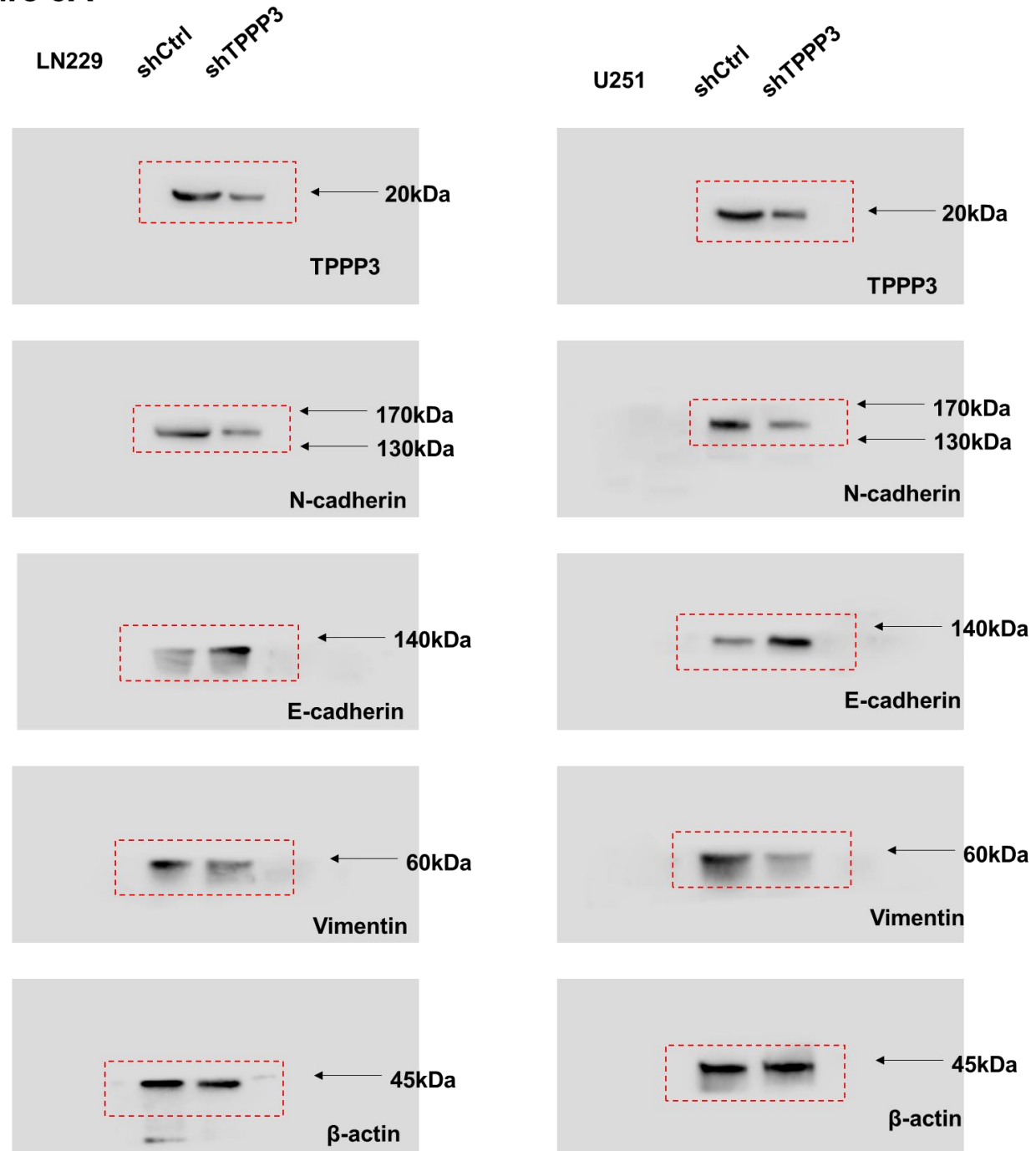

**Figure 4A**

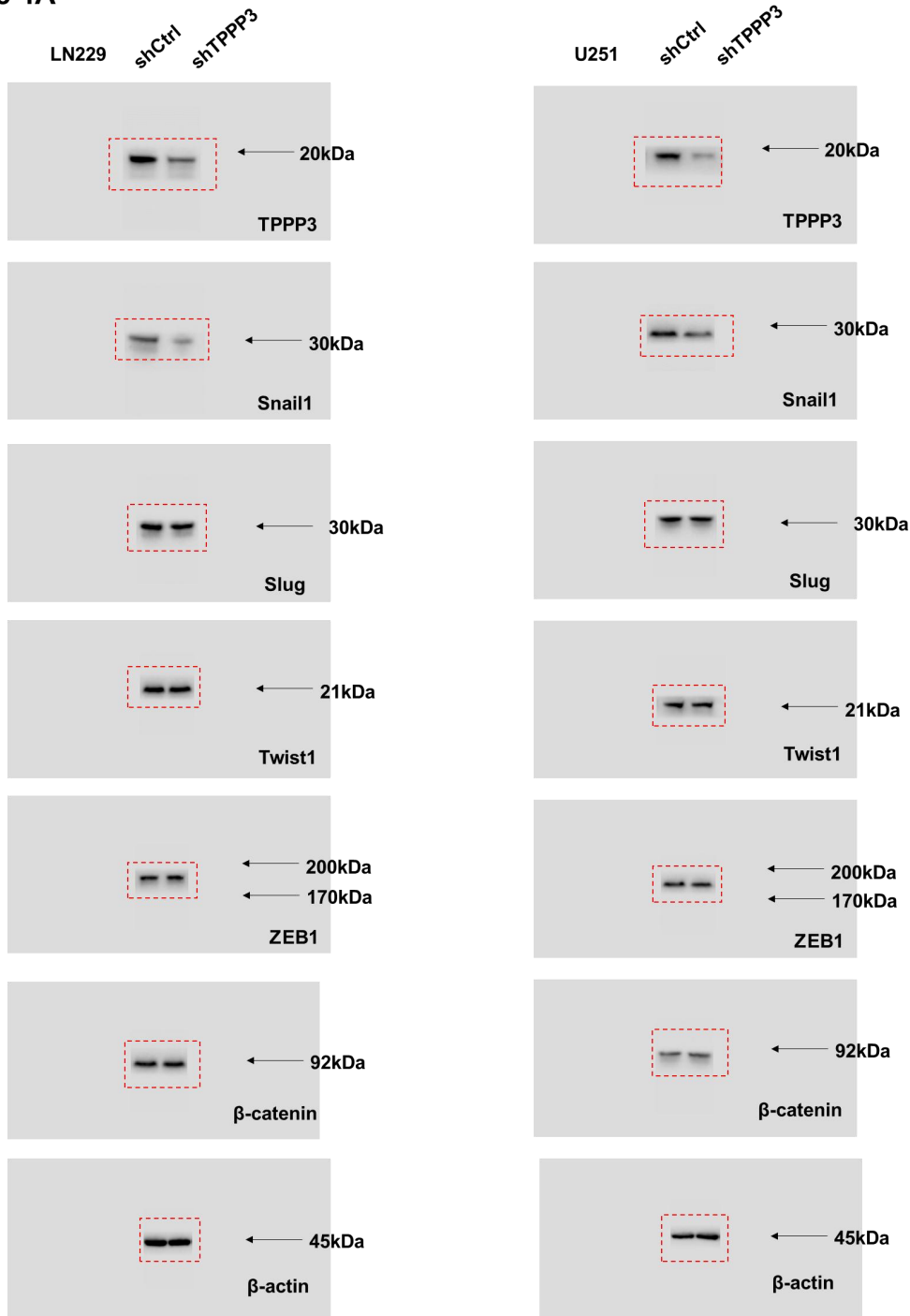

**Figure 4B**

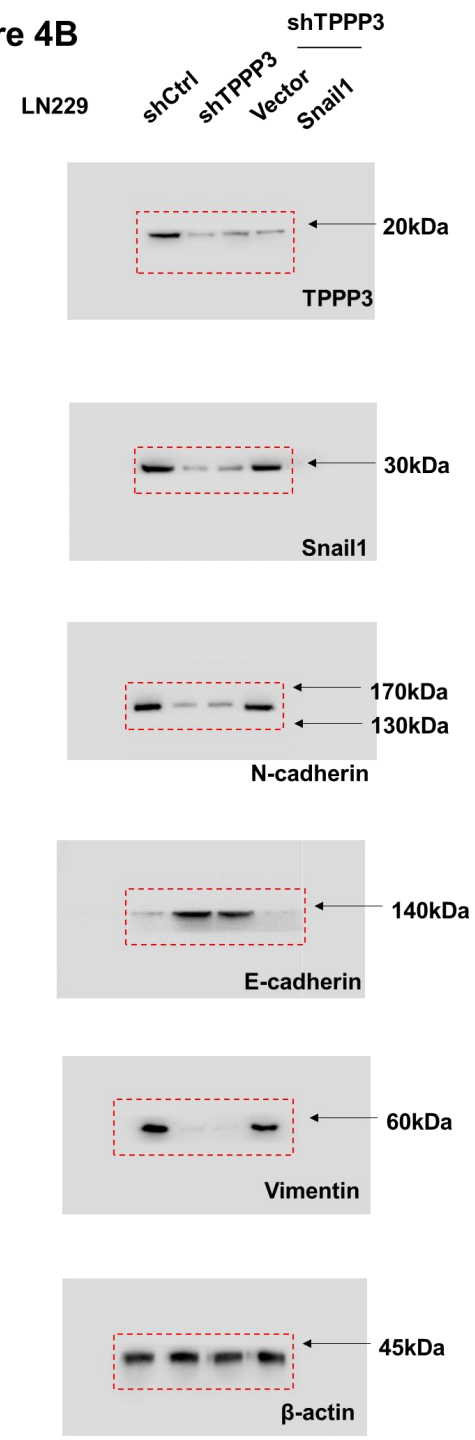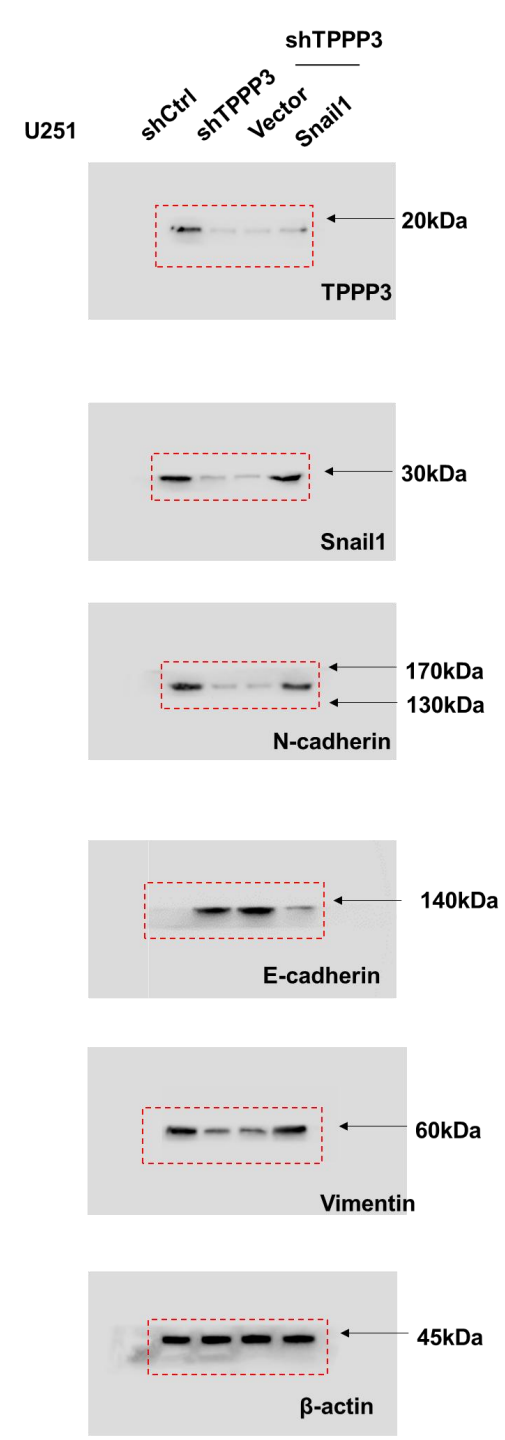

Supplement: Supplementary file 2 — Supplementary Figures. [file 41598_2023_45233_MOESM2_ESM.pdf]
